# D-Mannose Plus *Saccharomyces boulardii* to Prevent Urinary Tract Infections and Discomfort after Cystoscopy: A Single-Center Prospective Randomized Pilot Study

**DOI:** 10.3390/medicina59061165

**Published:** 2023-06-17

**Authors:** Carmelo Quattrone, Celeste Manfredi, Luigi Napolitano, Angelo Ferraro, Concetta Distefano, Antonio Di Girolamo, Carmine Sciorio, Vittorio Imperatore, Francesco Bottone, Roberto La Rocca, Davide Arcaniolo, Marco De Sio, Lorenzo Spirito

**Affiliations:** 1 Urology Unit, Department of Woman, Child and General and Specialized Surgery, University of Campania “Luigi Vanvitelli”, 80131 Naples, Italy; carmeloquattrone@hotmail.it (C.Q.); manfredi.celeste@gmail.com (C.M.); bottonefrancesco@yahoo.it (F.B.); davide.arcaniolo@gmail.com (D.A.); marco.desio@unicampania.it (M.D.S.); lorenzospirito@msn.com (L.S.); 2 Department of Neurosciences, Reproductive Sciences and Odontostomatology, University of Naples “Federico II”, 80131 Naples, Italy; robertolarocca87@gmail.com; 3 Urology Unit, AORN Moscati, 83100 Avellino, Italy; angelo86ferr@gmail.com (A.F.); antonio.digirolamo@hotmail.com (A.D.G.); v.imperatore1@gmail.com (V.I.); 4 Division of Urology, IRCCS Azienda Ospedaliero Universitaria di Bologna, 40138 Bologna, Italy; concettadistefano90@gmail.com; 5 Urology Unit, Ospedale Alessandro Manzoni, 23900 Lecco, Italy; carmine.sciorio@gmail.com

**Keywords:** cystoscopy, D-mannose, infection, probiotic, *Saccharomyces boulardii*, supplement

## Abstract

*Background and Objectives*: Patients undergoing cystoscopy can experience discomfort or pain during the procedure. In some cases, a urinary tract infection (UTI) with storage lower urinary tract symptoms (LUTS) may occur in the days following the procedure. This study aimed to assess the efficacy of D-mannose plus *Saccharomyces boulardii* in the prevention of UTIs and discomfort in patients undergoing cystoscopy. *Materials and Methods*: A single-center prospective randomized pilot study was conducted between April 2019 and June 2020. Patients undergoing cystoscopy for suspected bladder cancer (BCa) or in the follow-up for BCa were enrolled. Patients were randomized into two groups: D-Mannose plus *Saccharomyces boulardii* (Group A) vs. no treatment (Group B). A urine culture was prescribed regardless of symptoms 7 days before and 7 days after cystoscopy. The International Prostatic Symptoms Score (IPSS), 0–10 numeric rating scale (NRS) for local pain/discomfort, and EORTC Core Quality of Life questionnaire (EORTC QLQ-C30) were administered before cystoscopy and 7 days after. *Results*: A total of 32 patients (16 per group) were enrolled. No urine culture was positive in Group A 7 days after cystoscopy, while 3 patients (18.8%) in Group B had a positive control urine culture (*p* = 0.044). All patients with positive control urine culture reported the onset or worsening of urinary symptoms, excluding the diagnosis of asymptomatic bacteriuria. At 7 days after cystoscopy, the median IPSS of Group A was significantly lower than that of Group B (10.5 vs. 16.5 points; *p* = 0.021), and at 7 days, the median NRS for local discomfort/pain of Group A was significantly lower than that for Group B (1.5 vs. 4.0 points; *p* = 0.012). No statistically significant difference (*p* > 0.05) in the median IPSS-QoL and EORTC QLQ-C30 was found between groups. *Conclusions*: D-Mannose plus *Saccharomyces boulardii* administered after cystoscopy seem to significantly reduce the incidence of UTI, the severity of LUTS, and the intensity of local discomfort.

## 1. Introduction

Bladder cancer (BCa) is one of the most common cancers worldwide, with over 550,000 new cases diagnosed in 2020 [1]. Cystoscopy represents an essential tool for the diagnosis and follow-up of BCa [2,3]. Despite technological improvements that have made it a minimally invasive procedure, patients can experience discomfort or pain during the procedure, which may persist in the following days. Burning during urination and gross hematuria are common complaints after cystoscopy. In some cases, a urinary tract infection (UTI) with storage lower urinary tract symptoms (LUTS) and/or fever may occur in the days following the procedure [4]. However, no antibiotic prophylaxis is recommended by the current European Association of Urology (EAU) guidelines [5].

D-Mannose is a natural monosaccharide, normally involved in the glycosylation of proteins [6]. It may inhibit bacterial adhesion to urothelial cells, a crucial mechanism for the onset of infections and inflammation. More specifically, D-Mannose can hinder the adhesion of *Escherichia coli* through the inhibition of FimH adhesin, located at the tip of its type 1 fimbriae. As the antibiotic therapeutic strategy against urinary tract infections has become less effective due to the increase in resistance, and is frequently associated with multiple side effects, the antiadhesive molecules active against fimbrial adhesins appear today as an attractive alternative. FimH, generated by uropathogenic *Escherichia coli* (UPEC), is the best-characterized bacterial adhesin. Although these competitive antagonists preventing pathogen adhesion seem to be promising antimicrobial agents, the molecular mechanisms underlying these complex adhesion processes are still poorly understood [7,8]. Several studies have provided evidence supporting the efficacy of D-Mannose in the prevention and treatment of UTIs [7,9,10]. A recent review by De Nunzio et al. reported that most of the papers available on the topic demonstrate that D-Mannose can play a role in the prevention of recurrent UTIs or iatrogenic UTIs (i.e., urodynamics-associated), and its effect, in some cases, can be comparable to that of antibiotics; several articles proved that the combination of D-Mannose with other substances (including probiotics) could enhance efficacy in preventing UTIs without compromising patient safety [7]. 

*Saccharomyces boulardii* is a probiotic yeast commonly used in clinical practice for gastrointestinal disorders. Several studies in children have highlighted its potential role in preventing UTIs [11,12,13]. Consequently, the possible application of *Saccharomyces boulardii* in adult UTIs is conceivable; however, there is no research yet available on the topic.

The aim of the present study was to evaluate the efficacy of D-Mannose plus *Saccharomyces boulardii* in the prevention of UTIs and discomfort in patients undergoing cystoscopy.

## 2. Materials and Methods

### 2.1. Study Design and Ethical Details

We designed a single-center prospective randomized pilot study at the Urology Unit of the University of Campania “Luigi Vanvitelli” (Naples, Italy) that was conducted between April 2019 and June 2020. No placebo or blinding was applied. The study was approved by the local Ethics Committee. The research was conducted according to the Declaration of Helsinki on the ethical principles for medical research involving human subjects [14]. All patients provided written informed consent for study inclusion and data publication.

### 2.2. Patient Enrollment

Patients undergoing cystoscopy for suspected BCa or follow-up after transurethral resection of the bladder (TURB), with negative urine culture, were enrolled. The exclusion criteria were: bladder catheter present or removed in the last 30 days; bladder stones; diagnostic or therapeutic endoscopic procedures on the urinary tract (including TURB) in the last 3 months; bladder biopsy during cystoscopy; intravesical therapy for BCa in the last 30 days; history of interstitial cystitis, recurrent UTIs, or chronic prostatitis; presence of urethral strictures, meatal stenosis, urinary incontinence, neurogenic bladder, or severe phimosis; history of carcinoma in situ or muscle invasive tumors not undergoing radical cystectomy; immunodeficiency disorders; uncompensated diabetes mellitus (HbA1c > 7%); allergy to substances evaluated in the study; and difficulty swallowing tablets.

### 2.3. Treatment Protocol

All cystoscopies were performed by the same expert operator using a 16-Ch flexible cystoscope equipped with a sterile disposable sheath (Vision^®^ Sciences Inc., Orangeburg, NY, USA). No antibiotic prophylaxis was prescribed and 1% of lidocaine was applied into the urethra before each procedure. The bag squeeze technique was used in male patients for the passage of the instrument into the proximal urethra. Patients meeting the inclusion criteria were divided into two treatment groups based on a simple randomization performed with a computer-generated random list with a 1:1 ratio. According to this method, D-Mannose plus *Saccharomyces boulardii* (Group A) or no treatment (Group B) were prescribed after cystoscopy. Group A took one tablet every 12 h for 6 days starting on the day of cystoscopy. Each tablet contained D-Mannose (500 mg) and *Saccharomyces boulardii* (3.0 billion CFU). Adequate daily hydration was recommended to both groups in the week following cystoscopy. The use of other supplements, anti-inflammatory drugs, or antibiotics after cystoscopy was prohibited among all enrolled patients.

### 2.4. Patient Evaluation

Demographic data and clinical characteristics were collected for each patient at baseline. A urine culture was prescribed regardless of symptoms 7 days before and 7 days after cystoscopy. The urine culture results were sent by email. The urine culture before cystoscopy had to be negative to allow patients to be included in the study, while the urine culture result after cystoscopy was chosen as the primary outcome. The International Prostatic Symptoms Score (IPSS) [15], 0–10 numeric rating scale (NRS) for local pain/discomfort [16], and EORTC Core Quality of Life questionnaire (EORTC QLQ-C30) [17] were administered before cystoscopy in person and 7 days after by telephone and were selected as secondary outcomes.

### 2.5. Statistics

The continuous variables were described as medians and the interquartile range (IQR), while the categorical variables were expressed as frequencies and percentages. The Shapiro–Wilk test was applied as a normality test. The Wilcoxon rank-sum and Wilcoxon signed-rank tests were used to analyze continuous variables in independent and related samples, respectively. McNemar and Chi-squared tests were applied to analyze categorical variables in paired and unpaired samples, respectively. *p* < 0.05 was arbitrarily set to indicate statistical significance. The IBM Statistical Package for the Social Sciences (IBM Corp. Released 2017. IBM SPSS Statistics for Windows, Version 25.0. Armonk, NY, USA: IBM Corp.) was used for statistical analyses. G*Power (Heinrich-Heine-Universität Düsseldorf, Germany) was applied for the statistical power analysis, noting that a total sample size of 92 cases (46:46) was needed for a power of 0.80 (α = 0.05; β = 0.2) relative to the primary outcome.

## 3. Results

**Figure 1:**
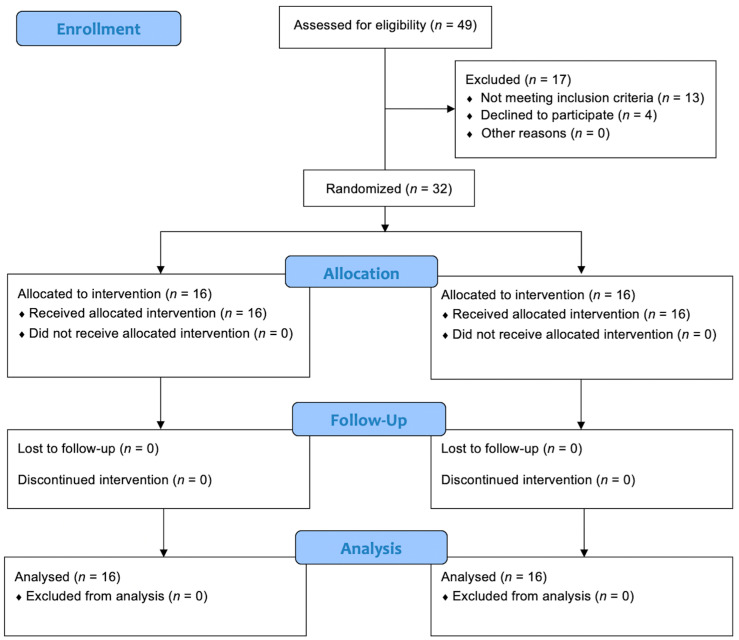
CONSORT flow diagram.

**Figure 2:**
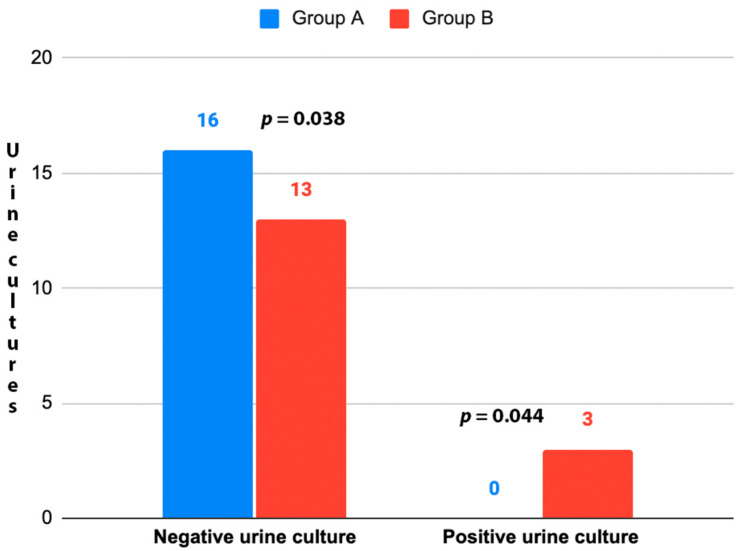
UTIs 7 days after cystoscopy. Group A: D-Mannose (500 mg) plus *Saccharomyces boulardii* (3.0 billion CFU); Group B: No treatment. UTI: Urinary Tract Infection. Chi-squared test used for the analyses.

A total of 32 patients were enrolled in the study and randomized into the two treatment groups. No patients or data were lost after enrollment (Figure 1). The baseline characteristics of the patients were reported in Table 1. No statistically significant differences (*p* > 0.05) between the two groups were found at baseline for the characteristics recorded. During the cystoscopies, no urethral strictures were found and no significant urethral trauma occurred. Mild gross hematuria was recorded in 4 patients (25.0%) in Group A and 5 (31.3%) in Group B. In all cases, it resolved without any additional maneuvers or treatments. Cystoscopy revealed the presence of suspicious lesions in 3 patients (18.8%) in Group A and 2 (12.5%) in Group B. Subsequent TURB showed that they were all low-grade Ta urothelial carcinomas. No urine culture was positive in Group A 7 days after cystoscopy, while 3 patients (18.8%) in Group B had a positive control urine culture (*p* = 0.044) (Figure 2). All positive urine cultures recorded the presence of *Escherichia coli*. All patients with positive control urine culture reported the onset or worsening of LUTS, excluding the diagnosis of asymptomatic bacteriuria. The median IPSS at baseline and 7 days after cystoscopy were not significantly different in Group A (8.0 vs. 10.5 points; *p* = 0.065), while a significant worsening of the median IPPS compared to baseline was recorded 7 days after cystoscopy in Group B (8.0 vs. 16.5 points; *p* ≤ 0.001). At 7 days after cystoscopy, the median IPSS of Group A was significantly lower than the median IPSS of Group B (10.5 vs. 16.5 points; *p* = 0.021). Similar results were found when analyzing NRS for local pain/discomfort; in fact, 7 days after cystoscopy, Group A reported a significantly lower median NRS than Group B (1.5 vs. 4.0 points; *p* = 0.012). No statistically significant difference (*p* > 0.05) in the median IPSS-QoL and EORTC QLQ-C30 was found when comparing patients of the same group between baseline and control time or comparing patients of Group A and Group B at 7 days after cystoscopy. The secondary outcomes are detailed in Table 2.

## 4. Discussion

Cystoscopy, a widely used diagnostic procedure in urology, can be frustrating for patients and is associated with possible infectious complications [4]. Post-cystoscopy UTIs can be very common, although the true prevalence is difficult to estimate due to mildly symptomatic patients in whom urine cultures are often not performed, or subjects treated empirically without urine culture by general practitioners [18]. For this reason, the prescription of antibiotic prophylaxis before cystoscopy remains a matter of debate. The current EAU guidelines recommend avoiding antibiotic prophylaxis in patients undergoing cystoscopy [5]; however, some evidence pushes in the opposite direction. Zeng et al., in a recent Cochrane meta-analysis on 20 randomized controlled clinical trials (RCTs) and 2 quasi-RCTs including 7711 patients, found that antibiotic prophylaxis may reduce the risk of symptomatic UTI (RR 0.49, 95% CI 0.28 to 0.86) without increasing serious and mild complications [19].

Obviously, the uncertainty on the topic and the concern about the resistance and possible adverse effects of antibiotics has prompted the search for possible alternatives to antibiotic prophylaxis. D-Mannose seems effective in the prevention and resolution of bladder inflammation and infection, representing a possible option in patients undergoing cystoscopy [7,18]. It is important to underline that the studies on D-Mannose in the cystoscopy setting are not available in the literature; therefore, the rationale for its use in this clinical context can only be inferred and speculated on the basis of previous articles investigating its role in the prevention or treatment of other UTIs. Salvatore et al. recently reported the results of a single-center, randomized, double-blind, placebo-controlled trial evaluating the safety and efficacy of a D-Mannose-based dietary supplement (D-Mannose, citric acid, prebiotic fibers, Astragalus, and dandelion [DAPAD complex]) for the treatment of uncomplicated acute *Escherichia coli* UTIs in non-menopausal women. Clinical resolution was higher in the DAPAD group compared to the placebo at 6 days (34.3% vs. 0%; *p* < 0.0001) and 35 days (88.6% vs. 20%, *p* < 0.0001). At day 35, no patients in the DAPAD group had moderate or severe symptoms, whereas 25.7% and 11.4% of patients in the placebo group had moderate and severe symptoms, respectively. Bacteriological resolution was also higher in the DAPAD group at day 6 (85.7% vs. 14.3%; *p* < 0.0001) and day 35 (100% vs. 40%; *p* < 0.0001). Only few mild adverse events (4.26%) unrelated to the investigated product were recorded [10]. Pugliese et al. investigated in a pilot study on 33 patients if the combination of D-Mannose, pomegranate extract, prebiotics, and probiotics was effective in modifying the symptoms reported by women with acute uncomplicated acute cystitis. At T1 (15 days), all symptoms or the majority of symptoms had disappeared in 10 women (30.3%), and at T2 (30 days), they had disappeared in 30 women (90.9%). The mean score reported at all the Acute Cystitis Symptom Score (ACSS) sub-scales significantly decreased between baseline and T1 and T2. No adverse events were recorded [9]. Despite these encouraging findings, the evidence on D-Mannose in the UTIs clinical setting remains somewhat controversial. Cooper et al., in a Cochrane review, analyzed 7 RCTs including 719 participants (adult females and males with acute cystitis or a history of recurrent UTIs), concluding that there is currently little to no evidence to support or refute the use of D-Mannose to prevent or treat UTIs in all populations [20].

On the other hand, *Saccharomyces boulardii* is a yeast commonly used in clinical practice for various gastrointestinal disorders accompanied by gut dysbiosis. Some evidence in children suggested the possible role of *Saccharomyces boulardii* for UTI prevention in the adult population [13]. Similarly to D-Mannose, there are no previous studies on the use of this yeast in the context of cystoscopy; therefore, the rationale for its use in this clinical setting can only be speculated from previous articles. Akil et al. evaluated the influence of oral *Saccharomyces boulardii* intake on the number of *Escherichia coli* colonies in the colons of 24 children. The mean number of colonies in g/mL stool decreased significantly with the treatment (384,625 ± 445,744 vs. 6283 ± 20,283; *p* = 0.00). [11]. Madden-Fuentes et al. assessed the ability of a fluoroquinolone-probiotic combination (ciprofloxacin + *Saccharomyces boulardii*) to prevent recurrent UTIs in 10 children. The authors found a significant decrease in the total number of UTI episodes in all patients compared before and after initiation therapy (57 vs. 4; *p* = 0.0001). Seven patients (70%) were free of recurrent UTIs during the follow-up period, and considering the patients with known compliance, 7 out of 8 were free of recurrent UTIs (88%) [12]. Despite these preliminary promising findings on children, it is clear that the evidence on *Saccharomyces boulardii* in the UTIs setting remains currently very limited.

Several other supplements have been evaluated over the years to reduce the inflammation and risk of infection of the lower urinary tract in various clinical settings [21,22].

Our study evaluated the efficacy of a supplement based on D-Mannose and *Saccharomyces boulardii* against UTIs and discomfort in patients undergoing cystoscopy. We found that this combination significantly reduced the risk of UTIs after cystoscopy. Furthermore, it was associated with lower LUTS and local discomfort than with no treatment. To the best of our knowledge, this is the first study evaluating the impact of a D-Mannose and *Saccharomyces boulardii* supplement on post-cystoscopy complications and symptoms. It also shows the advantage of being based on a prospective randomized design and validated questionnaires. Promising findings emerge from our research, but they should be read and interpretated according to several limitations. The small sample size (underpowered study) and short follow-up are the main issues, and they may have altered or prevented some results from being found. However, this study was designed as a pilot project on which to base future research with a larger sample size and longer follow-up. The absence of a placebo and blinding are additional weaknesses of our paper.

## 5. Conclusions

In conclusion, D-Mannose plus *Saccharomyces boulardii* administered after cystoscopy seem to significantly reduce the incidence of UTI, the severity of LUTS, and the intensity of local discomfort. Further studies with larger sample sizes and longer follow-ups are needed to confirm our encouraging results.

## Figures and Tables

**Table 1:** Baseline characteristics of patients.

	Group A (*n* = 16)	Group B (*n* = 16)	*p*-Value
Gender, *n* (%)			1.0
• Male	13 (81.3)	13 (81.3)	
• Female	3 (18.7)	3 (18.7)	
Age, *years* Median (IQR)	64.0 (55.5–70.0)	62.5 (53.5–68.0)	0.456
Obesity, *n (%)*	4 (25.0)	3 (18.8)	0.073
Diabetes mellitus, *n (%)*	2 (12.5)	2 (12.5)	1.0
BPH, *n (%)*	9 (56.2)	8 (50.0)	0.288
No history of BCa, *n (%)*	2 (12.5)	3 (18.8)	0.126
Follow-up for BCa, *n (%)*	14 (87.5)	13 (81.3)	0.087
• Low-grade pTa	9 (56.3)	8 (50.0)	
• Low-grade pT1	1 (6.3)	2 (12.5)	
• High-grade pT1	4 (25.0)	3 (18.8)	
IPSS, *points* Median (IQR)	8.0 (5.0–14.0)	8.0 (6.5–15.0)	0.359
IPSS-QoL, *points* Median (IQR)	2.0 (1.0–3.0)	2.5 (1.0–3.5)	0.232
0–10 NRS, *points* Median (IQR)	2.5 (1.0–4.0)	1.5 (1.0–3.0)	0.188
EORTC QLQ-C30, *points* Median (IQR)	50.0 (18.0–65)	48.0 (16.5–69)	0.071

Group A: D-Mannose (500 mg) plus *Saccharomyces boulardii* (3.0 billion CFU) vs. Group B: No treatment. BPH: Benign Prostatic Hypertrophy; EORTC Core Quality of Life questionnaire: EORTC QLQ-C30; IPSS: International Prostatic Symptoms Score; NRS: Numeric Rating Scale; QoL: Quality of Life. Wilcoxon rank-sum test used for continuous variables; Chi-squared test used for categorical variables.

**Table 2:** Evaluation of secondary outcomes.

	Group A (*n* = 16)		Group B (*n* = 16)		*p*-Value
	Baseline	At 7 Days	Baseline	At 7 Days	
IPSS, *points* Median (IQR)	8.0 (5.0–14.0)	10.5 (7.5–16.0)	8.0 (6.5–15.0)	16.5 (10.0–22.0)	0.065 ^a^ <0.001 ^b^ 0.021 ^c^
IPSS-QoL, *points* Median (IQR)	2.0 (1.0–3.0)	2.0 (1.5–2.5)	2.5 (1.0–3.5)	3.5 (2.0–4.0)	0.341 ^a^ 0.063 ^b^ 0.082 ^c^
0–10 NRS, *points* Median (IQR)	2.5 (1.0–4.0)	1.5 (1.0–4.5)	1.5 (1.0–3.0)	4.0 (2.5–5.0)	0.256 ^a^ <0.001 ^b^ 0.012 ^c^
EORTC QLQ-C30, *points* Median (IQR)	50.0 (18.0–65)	47.5 (19.0–62.5)	48.0 (16.5–69.0)	51.5 (18.0–68.5)	0.451 ^a^ 0.293 ^b^ 0.387 ^c^

Group A: D-Mannose (500 mg) plus *Saccharomyces boulardii* (3.0 billion CFU) vs. Group B: No treatment. ^a^ Group A at baseline vs. Group A at 7 days; ^b^ Group B at baseline vs. Group B at 7 days; ^c^ Group A at 7 days vs. Group B at 7 days. EORTC Core Quality of Life questionnaire: EORTC QLQ-C30; IPSS: International Prostatic Symptoms Score; NRS: Numeric Rating Scale; QoL: Quality of Life. Wilcoxon rank-sum and Wilcoxon signed-rank tests used for independent and related samples, respectively.

## Data Availability

Not applicable.

## References

[1] Saginala K., Barsouk A., Aluru J.S., Rawla P., Padala S.A., Barsouk A. (2020). Epidemiology of Bladder Cancer. Med. Sci..

[2] Turco C., Collà Ruvolo C., Cilio S., Celentano G., Califano G., Creta M., Capece M., La Rocca R., Napolitano L., Mangiapia F. (2022). Looking for cystoscopy on YouTube: Are videos a reliable information tool for internet users?. Arch. Ital. Urol. Androl..

[3] Capece M., Spirito L., La Rocca R., Napolitano L., Buonopane R., Di Meo S., Sodo M., Bracale U., Longo N., Palmieri A. (2020). Hexaminolevulinate blue light cystoscopy (Hal) assisted transurethral resection of the bladder tumour vs. white light transurethral resection of the bladder tumour in non-muscle invasive bladder cancer (NMIBC): A retrospective analysis. Arch. Ital. Urol. Androl..

[4] Pavone-Macaluso M., Lamartina M., Pavone C., Vella M. (1992). The flexible cystoscope. Int. Urol. Nephrol..

[5] (2023). EAU Guidelines on Urological Infections.

[6] Hang J., Wang J., Lu M., Xue Y., Qiao J., Tao L. (2022). Protein O-mannosylation across kingdoms and related diseases: From glycobiology to glycopathology. Biomed. Pharmacother..

[7] De Nunzio C., Bartoletti R., Tubaro A., Simonato A., Ficarra V. (2021). Role of D-Mannose in the Prevention of Recurrent Uncomplicated Cystitis: State of the Art and Future Perspectives.

[8] Wagenlehner F., Lorenz H., Ewald O., Gerke P. (2022). Why d-Mannose May Be as Efficient as Antibiotics in the Treatment of Acute Uncomplicated Lower Urinary Tract Infections—Preliminary Considerations and Conclusions from a Non-Interventional Study.

[9] Pugliese D., Acampora A., Porreca A., Schips L., Cindolo L. (2020). Effectiveness of a novel oral combination of D-Mannose, pomegranate extract, prebiotics and probiotics in the treatment of acute cystitis in women. Arch. Ital. Urol. Androl..

[10] Salvatore S., Ruffolo A.F., Stabile G., Casiraghi A., Zito G., De Seta F. (2023). A Randomized Controlled Trial Comparing a New D-Mannose-based Dietary Supplement to Placebo for the Treatment of Uncomplicated *Escherichia coli* Urinary Tract Infections. Eur. Urol. Focus..

[11] Akil I., Yilmaz O., Kurutepe S., Degerli K., Kavukcu S. (2006). Influence of oral intake of *Saccharomyces boulardii* on *Escherichia coli* in enteric flora. Pediatr. Nephrol..

[12] Madden-Fuentes R.J., Arshad M., Ross S.S., Seed P.C. (2015). Efficacy of Fluoroquinolone/Probiotic Combination Therapy for Recurrent Urinary Tract Infection in Children: A Retrospective Analysis. Clin. Ther..

[13] Forster C.S., Hsieh M.H., Cabana M.D. (2021). Perspectives from the Society for Pediatric Research: Probiotic use in urinary tract infections, atopic dermatitis, and antibiotic-associated diarrhea: An overview. Pediatr. Res..

[14] World Medical Association (2013). World Medical Association Declaration of Helsinki. Ethical principles for medical research involving human subjects. JAMA.

[15] Yao M.W., Green J.S.A. (2022). How international is the International Prostate Symptom Score? A literature review of validated translations of the IPSS, the most widely used self-administered patient questionnaire for male lower urinary tract symptoms. Low. Urin. Tract. Symptoms.

[16] Krebs E.E., Carey T.S., Weinberger M. (2007). Accuracy of the pain numeric rating scale as a screening test in primary care. J. Gen. Intern. Med..

[17] Niezgoda H.E., Pater J.L. (1993). A validation study of the domains of the core EORTC quality of life questionnaire. Qual. Life Res..

[18] Cusumano J.A., Hermenau M., Gaitanis M., Travis M., LaPlante K.L., Tran T.Y., McConeghy K.W. (2020). Evaluation of post-flexible cystoscopy urinary tract infection rates. Am. J. Health Syst. Pharm..

[19] Zeng S., Zhang Z., Bai Y., Sun Y., Xu C. (2019). Antimicrobial agents for preventing urinary tract infections in adults undergoing cystoscopy. Cochrane Database Syst. Rev..

[20] Cooper T.E., Teng C., Howell M., Teixeira-Pinto A., Jaure A., Wong G. (2022). D-mannose for preventing and treating urinary tract infections. Cochrane Database Syst. Rev..

[21] Manfredi C., Spirito L., Calace F.P., Balsamo R., Terribile M., Stizzo M., Romano L., Napolitano L., Califano G., Cirillo L. (2022). Oral Preparation of Hyaluronic Acid, Chondroitin Sulfate, Curcumin, and Quercetin (Ialuril^®^ Soft Gels) for the Prevention of LUTS after Intravesical Chemotherapy. Pathophysiology.

[22] Manfredi C., Calace F.P., Fusco F., Quattrone C., Giordano D., Crocetto F., Creta M., De Sio M., Arcaniolo D. (2021). *Escherichia coli* Nissle 1917 as adjuvant therapy in patients with chronic bacterial prostatitis: A non-blinded, randomized, controlled trial. World J. Urol..

